# *Crmp4* deletion promotes recovery from spinal cord injury by neuroprotection and limited scar formation

**DOI:** 10.1038/srep08269

**Published:** 2015-02-05

**Authors:** Jun Nagai, Yoshiteru Kitamura, Kazuki Owada, Naoya Yamashita, Kohtaro Takei, Yoshio Goshima, Toshio Ohshima

**Affiliations:** 1 Department of Life Science and Medical Bioscience, Graduate School of Advanced Science and Engineering, TWIns, Waseda University, Tokyo, 162-8480 Japan; 2 Research Fellow of Japan Society for the Promotion of Science; 3 Department of Molecular Pharmacology and Neurobiology, Graduate School of Medicine, Yokohama City University, Yokohama 236-0004, Japan; 4 Department of Medical Life Science, Graduate School of Medical Life Science, Yokohama City University, Yokohama, 236-0004, Japan

## Abstract

Axonal outgrowth inhibitors and scar formation are two major obstacles to central nervous system (CNS) repair. No target molecule that regulates both axonal growth and scarring has been identified. Here we identified collapsin response mediator protein 4 (CRMP4), a common mediator of inhibitory signals after neural injury, as a crucial factor that contributes to both axonal growth inhibition and scarring after spinal cord injury (SCI). We found increases in the inhibitory and toxic forms of CRMP4 in injured spinal cord. Notably, CRMP4 expression was evident in inflammatory cells as well as in neurons after spinal cord transection. *Crmp4*−/− mice displayed neuroprotection against SCI and reductions in inflammatory response and scar formation. This permissive environment for axonal growth due to CRMP4 deletion restored locomotor activity at an unusually early phase of healing. These results suggest that deletion of CRMP4 is a unique therapeutic strategy that overcomes two obstacles to CNS repair after SCI.

Injured adult mammalian central nervous system (CNS) has limited regenerative ability. Recovery after CNS injury is potently restricted by two main obstacles: axon growth inhibitors and scar formation^1^,^2^,^3^. Myelin-associated inhibitors (MAIs), expressed in myelin sheaths surrounding CNS axons, are non-permissive as substrates for neurite outgrowth^4^,^5^. Within the scar tissue at the lesion, extracellular molecules such as members of the chondroitin sulfate proteoglycan (CSPG) family^2^,^6^,^7^ and repulsive guidance molecule Semaphorin3A (Sema3A)^8^,^9^ become evident after injury and inhibit the regenerative responses of axons. Although numerous reports have suggested a number of target molecules for CNS repair after traumatic injury^10^, no target molecule that regulates both axonal growth and scarring has been identified.

Cytoskeletal dynamics is a key factor limiting the regenerative capacity of the CNS in terms of axon formation, inflammation, and scarring^11^,^12^,^13^,^14^,^15^,^16^. Collapsin response mediator protein 4 (CRMP4) is one of the CRMP family of proteins that is highly expressed in the developing and adult nervous system among vertebrates, and regulates aspects of neurite growth by their binding to the cytoskeleton^17^,^18^. Sema3A-induced phosphorylation of CRMP4^19^,^20^ reduces its binding affinity for tubulin heterodimer and F-actin, resulting in cytoskeletal depolymerization^21^,^22^. We and another group have demonstrated that long-form CRMP4^23^ (CRMP4b) is required for inhibitory responses to MAIs *in vitro*^24^,^25^,^26^. This suggests the possibility of CRMP4 involvement in CSPG-induced signaling because MAIs and CSPG inhibition share intracellular mechanisms through their common receptor NgR^27^. Moreover, injury-induced neuronal calpain activation produces a C-terminus-truncated form of CRMP4 (tCRMP4) that initiates neuronal cell death^28^,^29^. CRMP4 is therefore a common mediator of several inhibitory signaling pathways operating after traumatic injury. However, the role of CRMP4 following a traumatic CNS injury *in vivo* has never been examined.

Here we characterized CRMP4 as a unique factor that is responsible for both axonal growth inhibition and scar formation after SCI. We found an increase in phosphorylated CRMP4 (pCRMP4), CRMP4b, and tCRMP4 in injured spinal cord. The *Crmp4*−/− mice recently generated by our group^30^ exhibited neuroprotective effects against SCI by suppressing depolymerization of microtubules, apoptosis, and demyelination. Notably, CRMP4 expression was upregulated in activated microglia/macrophage and reactive astrocytes after SCI, consistent with an observed reduction of inflammatory responses and scarring upon CRMP4 deletion. This provided a permissive environment for the growth of spinal axons and improved locomotion after SCI. Our results suggest that an inactivation of *Crmp4* is a potential therapeutic strategy that addresses two main obstacles to recovery after SCI.

## Results

### Increased expression levels of CRMP4 after SCI

**Figure 1:**
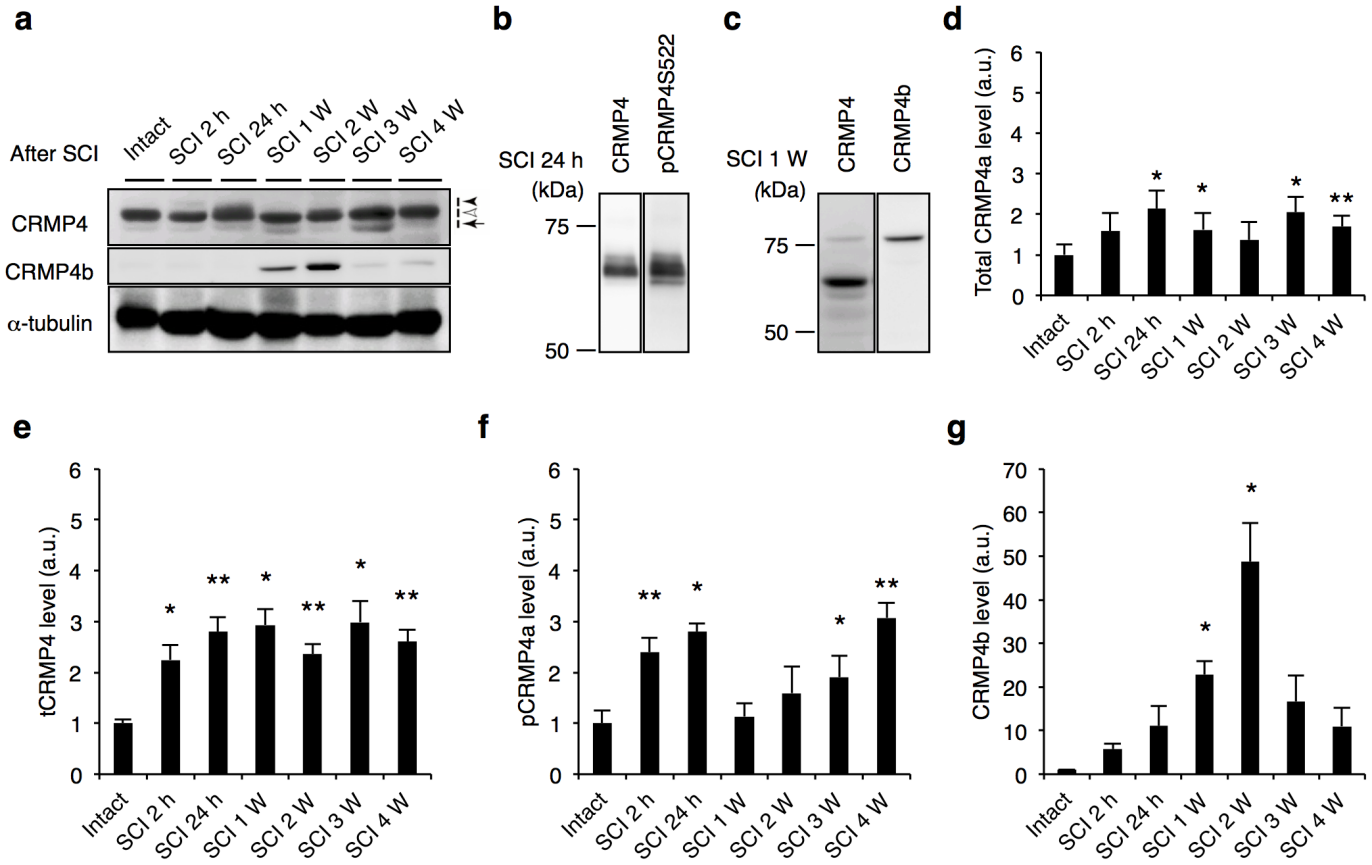
Change of CRMP4 expression level after SCI. (a) Immunoblot analysis of CRMP4 levels in intact and injured spinal cords from wild-type mice with anti-CRMP4 and anti-CRMP4b antibodies. Anti-CRMP4 antibody detected a part of phosphorylated CRMP4a (solid arrowhead), tCRMP4a (solid arrow) around the band of 65-kDa CRMP4a (open arrowhead). (b–c) Band pattern comparison between with anti-CRMP4 antibody and with the antibody for phosphorylated CRMP4 at Ser522 residue (pCRMP4S522) (b) and between anti-CRMP4 and anti-CRMP4b antibodies (c). (d–g) Quantitative analysis of different forms of CRMP4. Note the increase expression level of truncated form of CRMP4 and pCRMP4 and CRMP4b that are inhibitory to axonal growth. *, *P* < 0.05, **, *P* < 0.01. compared with the intact spinal cord. n = 5 mice per time-point. Statistical analysis was performed using one-way ANOVA followed by Dunnett's test. Data are mean ± S.E.M. h, hours; W, weeks.

In this study, we examined the role of CRMP4 in recovery after SCI. We first analyzed the change of CRMP4 protein expression after dorsal transection of the mouse spinal cord. To examine the temporal changes in the protein levels of CRMP4 and its phosphorylated and truncated forms after SCI, we performed immunoblotting of the spinal cord tissues at several time points post SCI. We detected three bands around the 65-kDa CRMP4a isoform with anti-CRMP4 antibody (Fig. 1a). The total amount of CRMP4a and the protein level of the 58-kDa tCRMP4 (Fig. 1a; solid arrow) were increased in injured spinal cords (Fig. 1d,e). The upper of the three bands (Fig. 1a; solid arrowhead) was identified as part of phosphorylated CRMP4 where the antibody is specific to CRMP4 phosphorylated at Ser522 (Fig. 1b). It was upregulated in both the acute and the sub-chronic phases of injury (Fig. 1f). Moreover, the 75-kDa isoform of CRMP4 was identified as CRMP4b using a specific antibody (Fig. 1c). Levels of CRMP4b were distinctly higher in injured spinal cords, peaking 1 to 2 weeks post SCI (Fig. 1a,g). These results indicate that forms of CRMP4 that are toxic or inhibitory to axonal growth were induced by SCI with a unique time course.

**Figure 2:**
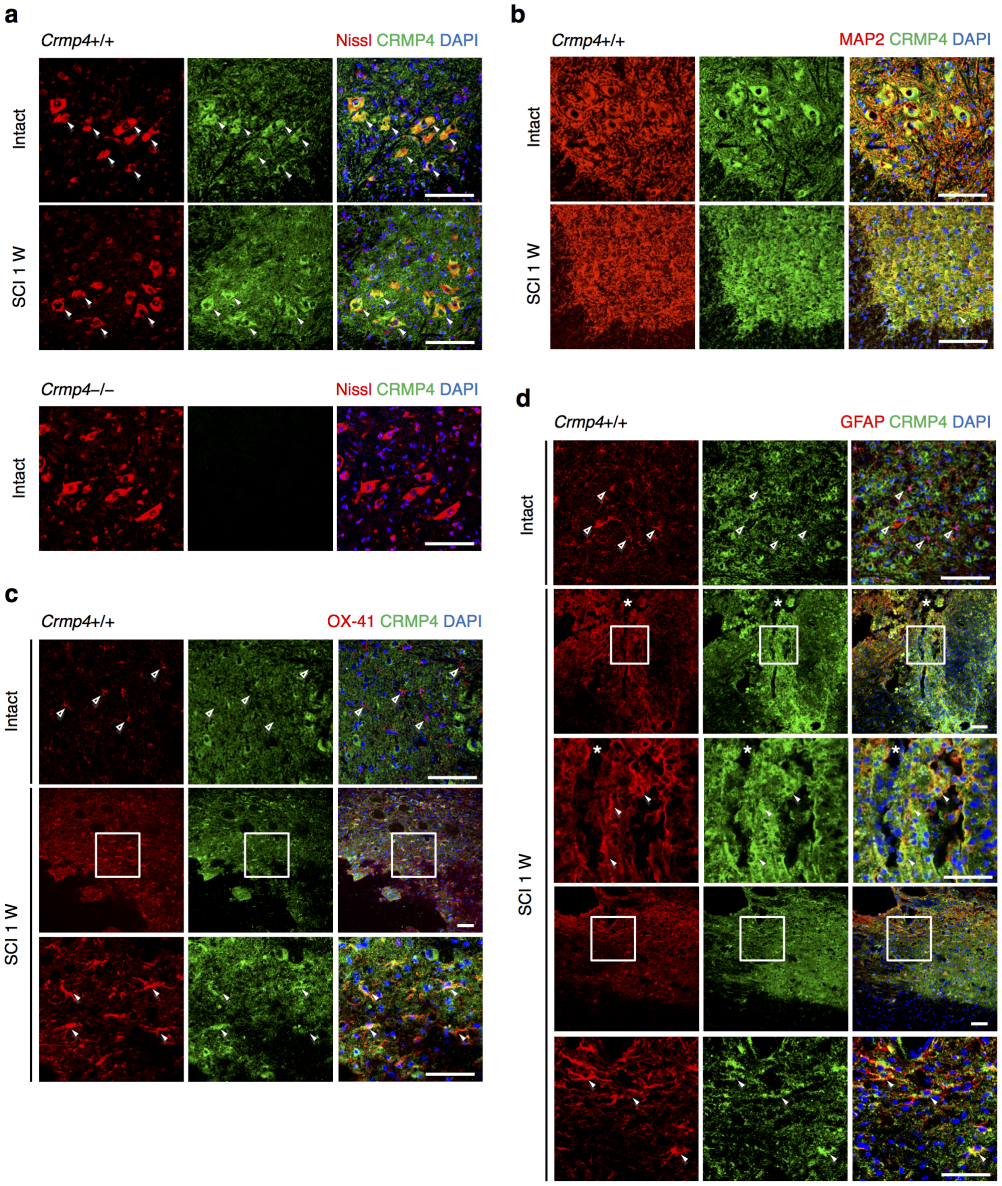
Expression changes of CRMP4 in spinal motoneuron and glial cells after SCI. Immunohistochemical analysis of the expression of CRMP4 of the intact and injured spinal cords at 1 week after transection (SCI 1 W). (a) Representative images from cross sections of spinal cord show double-immunofluorescent staining for Nissl (the marker for neurons; red) and CRMP4 (green). CRMP4 staining was apparent in Nissl-positive motoneuron in the ventral horn (solid arrowheads) both in intact and injured spinal cords from control *Crmp4*+/+ mice. This CRMP4 signal was undetectable in intact *Crmp4*−/− spinal cord. (b) Co-localization of CRMP4 (green) and MAP2 (red) immunopositive structures, which labeled neuronal cell bodies and their dendrites, in the ventral horn of intact and transected spinal cords. (c–d) Immunohistochemical analysis of the expression of CRMP4 in microglia/macrophage and astrocytes. (c) Images of sagittal sections show double immunofluorescent staining for CRMP4 (green) and red signals of OX-41, the marker for microglia/macrophage (c), or GFAP, the marker for normal and reactive astrocytes (d). In the intact spinal cord, red signals in the resting OX-41-positive microglia/macrophage and GFAP-positive astrocytes did not co-localized with green signals of CRMP4 (open arrowheads). However, at 1 week after SCI, CRMP4 signals are evident in activated these cells (arrowheads) adjacent lesion epicenter and astroglial scar. Nuclei were counterstained with DAPI (blue) in the same view in each section. Asterisks in d indicate lesion site. W, weeks. Scale bars: 100 μm.

Next, we examined which cell types expressed CRMP4 after SCI. A markedly increased CRMP4 expression level has been reported in spinal motoneurons in the mutant SOD1 mouse model^31^ and in adult sensory neurons after sciatic nerve injury^32^. We first conducted double immunostaining for neuronal marker and CRMP4 in cross sections of spinal cords. We detected CRMP4 expression and found that it was co-localized with Nissl-positive neuronal cell bodies and MAP2-positive dendrites and somata of motoneurons in the ventral horn of intact and injured spinal cords (Fig. 2a,b). The fraction of neurons expressing these levels of CRMP4 was significantly above background in *Crmp4*−/− spinal cords (Fig. 2a). To analyze CRMP4 protein expression in microglia/macrophages and reactive astrocytes before, during, and after inflammatory responses to SCI, we double immunostained parasagittal sections of spinal cords for CRMP4 and glial fibrillary acidic protein (GFAP), a marker for normal as well as reactive astrocytes, or OX-41, a marker for microglia/macrophages^33^. The CRMP4 staining signal was weak in both GFAP-positive and OX-41-positive cells in the gray matter of intact spinal cord (Fig. 2c,d; open arrowheads). However, CRMP4 immunoreactivity was remarkably enhanced in both types of glial cells adjacent to the lesion site and in the astroglial scar after SCI (Fig. 2c,d; solid arrowheads).

### Suppressed microtubule destabilization of axons in injured *Crmp4*−/− spinal cords

**Figure 3:**
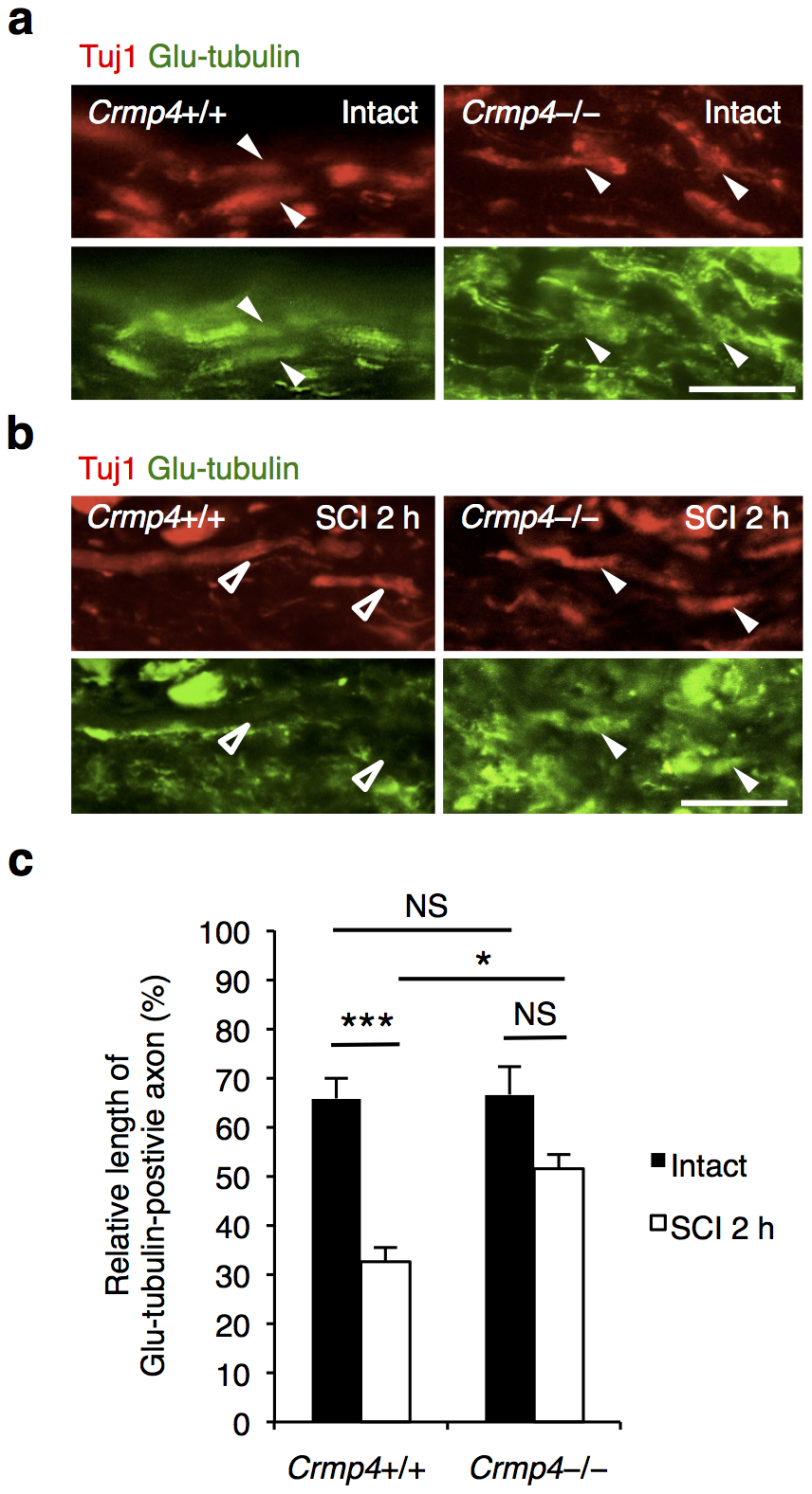
Suppression of microtubule depolymerization in the axons of the injured spinal cord on deletion of CRMP4. (a,b) Representative images of double immunohistochemistry for neuron-specific class III β-tubulin (red) and Glu-tubulin (green), which is abundant in polymerized microtubules. Glu-tubulin-positive stable microtubules showed a distribution along the Tuj1-positive axons in the white matter of intact spinal cords (a; arrowheads). This distribution was remarkably reduced at 2 hours after SCI (SCI 2 h) in *Crmp4*+/+ mice in the dorsal white matter (b; open arrowheads). However, the staining pattern of Glu-tubulin was significantly preserved in *Crmp4*−/− mice (b; arrowheads). Scale bar: 20 μm. (c) Quantification of relative length of Glu-tubulin-positive microtubules to Tuj1-positive axons in the dorsal white matter within 3 mm rostral and caudal to the lesion epicenter. n = 6 mice for each genotypes. *, *P* < 0.05, ***, *P* < 0.001. Statistical analysis was performed using one-way ANOVA followed by Tukey's multiple-comparison test. Data are mean ± S.E.M. h, hours; NS, not significant.

Given the increased expression of forms of CRMP4 inhibitory to axonal outgrowth observed after SCI, we next analyzed the effect of loss of function of CRMP4 on microtubule polymerization in injured axons in the acute phase of SCI. A previous study has shown that polymerized microtubule levels in white matter axons were decreased at 2 h post SCI^34^. We conducted immunostaining with anti-Glu-tubulin antibody to measure the polymerized forms of microtubules after SCI. In intact spinal cord, Glu-tubulin was present in a line along the Tuj1-positive axons in the white matter both in control *Crmp4*+/+ mice and in *Crmp4*−/− mice (Fig. 3a; arrowheads). At 2 h post SCI, the control mice showed a 50.7% reduction in the relative length of Glu-tubulin-positive axons in the dorsal white matter about 3 mm rostral to the injury site (Fig. 3b; open arrowheads). However, this decrease of microtubule polymerization in injured axons was reduced to 22.8% in *Crmp4*−/− mice (Fig. 3b, arrowheads; *P* < 0.05 compared with Fig. 3c, SCI 2 h control). This result indicates that deletion of CRMP4 contributes to stabilizing microtubules in the acute phase of SCI.

### CRMP4 in glial cells contributes to inflammatory response and scarring

To clarify the role of CRMP4 upregulation in activated microglia/macrophage and reactive astrocytes (Fig. 2c,d), we next assessed the degree of inflammation in *Crmp4*−/− mice after traumatic injury, which induces secondary tissue damage and scarring^35^.

First, we utilized a non-traumatic inflammation model to clarify the role of CRMP4 in the inflammatory response. Microinjections of Zymosan A into lateral white matter of thoracic spinal cord caused marked activation of Iba1-positive microglia/macrophages and GFAP-positive astrocytes at 3 days post injection in control mice (Supplementary Fig. S1a). These activations were undetectable around a PBS injection site, indicating that micropipette insertion was non-traumatic. In contrast, *Crmp4*−/− exhibited dramatic reduction in the area of inflammatory activation throughout the Zymosan A injection site (*P* < 0.05 compared with control mice, Supplementary Fig. S1a–c). Additionally, the protein expression level of Tumor necrosis factor αlπηα (TNFα) was elevated in Zymosan A-injected spinal cord of control mice when compared with PBS-injected control mice (*P* < 0.05, Supplementary Fig. S1d,e). This increase was significantly reduced by deletion of CRMP4 (*P* < 0.05 compared with Zymosan A-injected control mice, Supplementary Fig. S1d,e).

**Figure 4:**
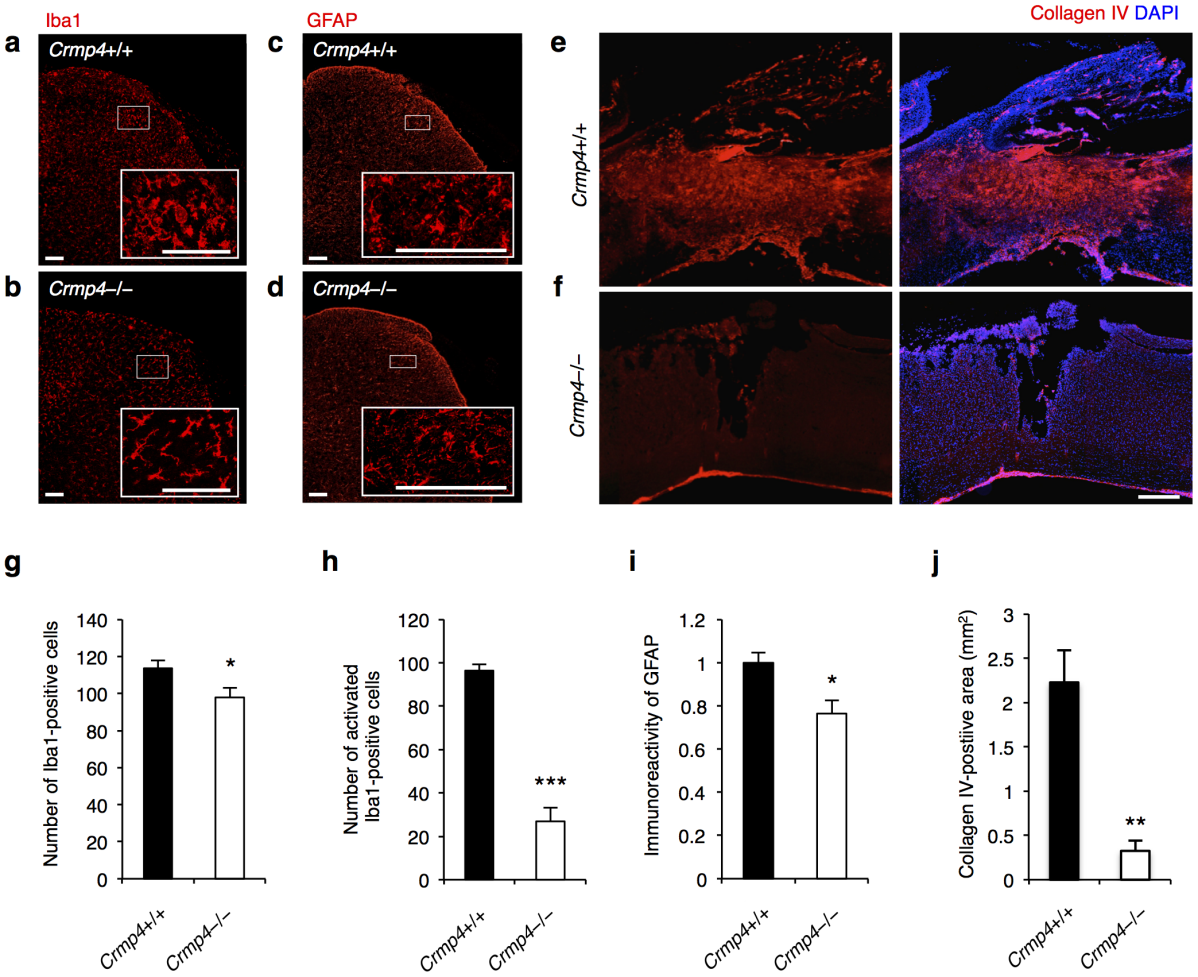
Deletion of CRMP4 suppresses inflammatory responses and scar formation after SCI. (a,b) Representative images of immunohistochemistry for Iba1, the marker for microglia/macrophage, at 1.5 mm caudal to tissue lesions at 1 week after SCI. While *Crmp4*+/+ mice with SCI demonstrated activated forms, determined by with longer processes than the soma diameter, *Crmp4*−/− mice exhibited resting morphology of microglia/macrophage. (c,d) GFAP staining revealed hypertrophic phenotype of reactive astrocytes in dorsal horn of injured spinal cords. Although no clear difference between *Crmp4*+/+ and *Crmp4*−/− spinal cords in terms of astrocyte morphological changes was observed, there seemed to be decreased immunoreactivity of GFAP signals in injured *Crmp4*−/− compared with controls. (e,f) Representative images of sagittal sections of collagen IV-positive scar formation at 1 week after SCI. (g–i) Quantifications of inflammatory responses in the dorsal horn at 1.5 mm caudal to injury site. *Crmp4*−/− mice exhibited decreased total number of Iba1-positive cells (g), reduced number of activated Iba1-positive cells (h) and suppressed immunoreactivity of GFAP (i) when compared with control mice (n = 5 mice for each genotypes). (j) Quantitative analysis of the area of collagen IV-positive scar tissue shows dramatic reduction of scarring in *Crmp4*−/− spinal cords compared with *Crmp4*+/+ one (n = 5 mice for each genotypes). *, *P* < 0.05, **, *P* < 0.01, ***, *P* < 0.001 compared with *Crmp4*+/+ controls. Statistical analysis was performed using an unpaired Student's *t* test. Data are mean ± S.E.M. Scale bars: 100 μm in a–d, 500 μm in e,f.

We next examined inflammatory responses in the spinal cord after SCI. Microglia/macrophage and astrocytes exhibit small, compact somata bearing many long, thin, ramified processes in their resting state. However, activated microglia/macrophage and reactive astrocytes demonstrate marked cellular hypertrophy and retraction of cytoplasmic processes^36^,^37^. At 1 week post SCI, microglia in the dorsal horn of control spinal cords at 1.5 mm caudal to lesion epicenter exhibited an activated phenotype (Fig. 4a). In contrast, *Crmp4*−/− mice showed moderate expression of resident microglia exhibiting the quiescent or resting type morphology in both white and gray matter of spinal cord (Fig. 4b). The numbers of Iba1-positive cells and activated Iba1-positive cells in dorsal horn of *Crmp4*−/− spinal cord were decreased 13.7% and 72.1%, respectively, when compared to control spinal cord (Fig. 4g, *P* < 0.05; Fig. 4h, *P* < 0.001). GFAP-positive astroglia demonstrating a swollen hypertrophic appearance were distributed throughout both white and gray matter in injured control spinal cords (Fig. 4c). Although CRMP4 deletion had no effect on levels of such astroglial morphology in the dorsal horn of injured spinal cord (Fig. 4d), *Crmp4*−/− spinal cord exhibited 23.6% reduction in immunoreactivity of GFAP in the dorsal horn when compared with control spinal cord (Fig. 4i, *P* < 0.05). These results indicate that the activation of microglia/macrophage and astrocytes observed in *Crmp4*+/+ mice after injury was minimal in *Crmp4*−/− mice.

Third, we assessed scar formation after SCI in *Crmp4*−/− mice. The lesion scar in traumatic SCI consists of a fibrous scar at the lesion core surrounded by a glial scar^2^. We observed a collagen IV-positive fibrous scar around the lesion epicenter in control spinal cord at 1 week post SCI (Fig. 4e). However, in *Crmp4*−/− spinal cords, an 85.4% reduction in collagen IV-positive scar was observed (Fig. 4f,j, *P* < 0.05 compared with control mice,). These findings indicate that CRMP4 deletion prevents scarring by moderating inflammatory responses after SCI.

### Neuroprotection at the lesion site in transected *Crmp4*−/− spinal cord

**Figure 5:**
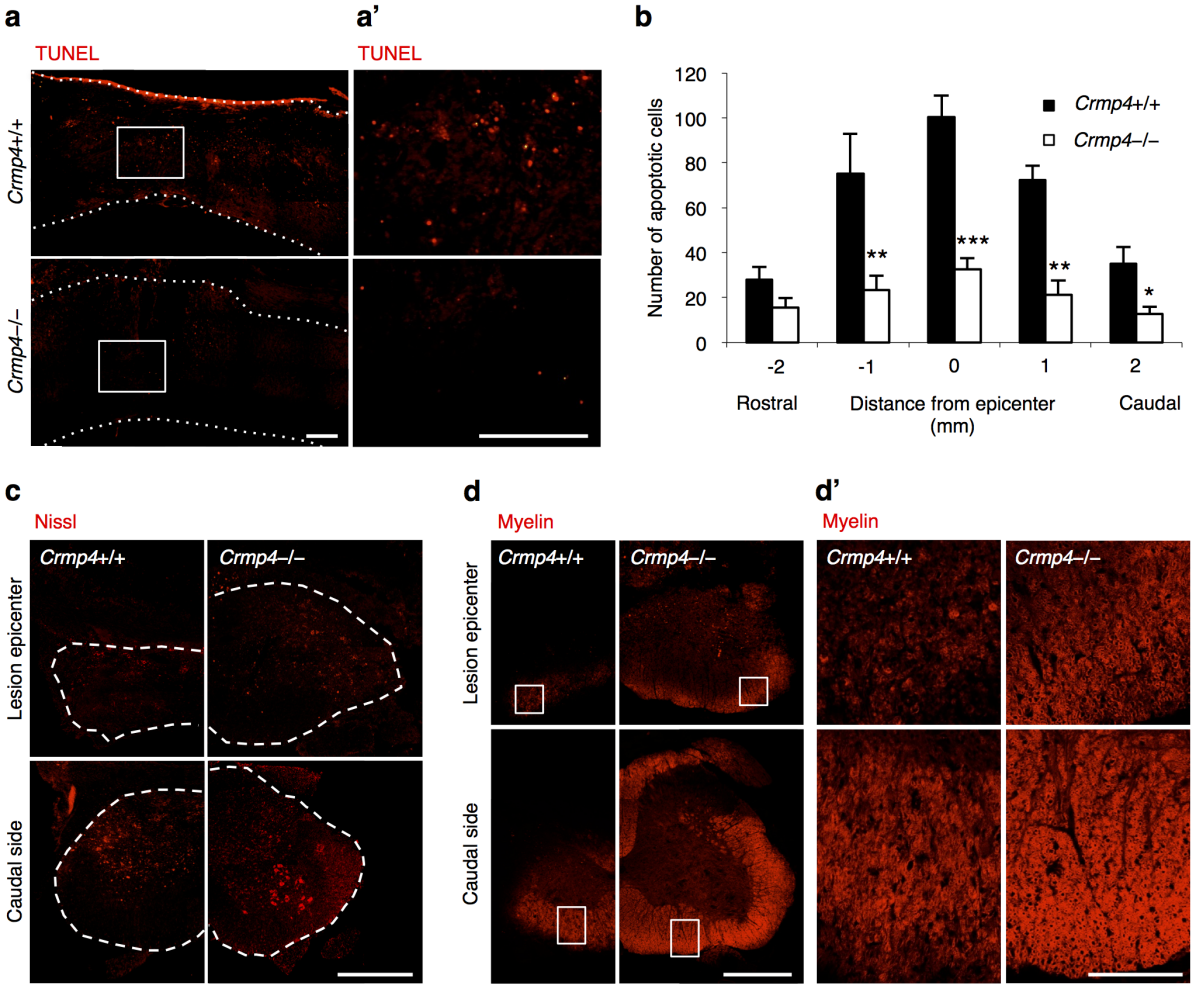
Decreased apoptotic cell death and demyelination in the injured spinal cord of *Crmp4*−/−mice. (a) Representative images of sagittal sections of TUNEL-positive apoptotic cells at 1 week post SCI. (a′) Magnified images of the indicated areas in a. (b) Quantitative analysis of the numbers of TUNEL-positive apoptotic cells showed decreased apoptotic cells in *Crmp4*−/− spinal cords when compared with those in *Crmp4*+/+ controls (*, *P* < 0.05, **, *P* < 0.01, ***, *P* < 0.001). Statistical analysis was performed using an unpaired Student's *t* test. n = 5 mice for each genotypes. Data are mean ± S.E.M. (c) Nissl staining in cross sections of the center and 1.5 mm caudal to injury site revealed increased cell survival in *Crmp4*−/− spinal cords when compared with in *Crmp4*+/+ controls at 4 weeks post SCI. (d) Images from cross sections of the center and 900 μm caudal to injury site with myelin staining. *Crmp4*−/− spinal cords showed higher density of myelin signals and larger are of white matter at both positions when compared with in *Crmp4*+/+ controls at 4 weeks post SCI. Scale bar: 100 μm in a,a′,d′, 500 μm in c,d.

Extensive inflammation-induced tissue injury causes impaired CNS function because of delayed secondary neuronal damage, such as neuronal loss and demyelination^38^,^39^. Additionally, it has been suggested that the calpain-mediated C-terminus truncation product of CRMP4 (tCRMP4) is associated with neuronal apoptosis after neurotoxin treatment^28^. To analyze the effect of CRMP4 deletion on cell survival in lesioned spinal cords, we performed the TUNEL assay (terminal deoxynucleotidyl transferase (TdT)-mediated deoxyuridine triphosphate-biotin nick end labeling) to detect apoptosis in both groups at 1 week post injury, a time point corresponding to the peak of secondary apoptosis at the lesion site^40^. *Crmp4*−/− mice showed 68.7%, 70.9%, 63.4% and 67.5% reductions in the number of TUNEL-positive apoptotic cells at 1 mm rostral to, 1 and 2 mm caudal to, and central to the lesion epicenter, respectively (*P* < 0.05 compared with control mice, Fig. 5a,b). Next, to examine the effect of CRMP4 deletion on tissue loss after SCI, we performed Nissl and myelin staining in serial cross sections through the lesion epicenter at 1 and 4 weeks post transection. Although differences in spinal tissue volume were not observed between *Crmp4*−/− spinal cord and control one at 1 week post SCI (Supplementary Fig. S2a, S3a), CRMP4 deletion resulted in tissue sparing around the lesion epicenter at 4 weeks post traumatic injury (Supplementary Fig. S2b, S3b). The *Crmp4*−/− spinal cord exhibited increased neuronal cell survival (Fig. 5c), spared white matter area, and reduced demyelination when compared with control spinal cords at 4 weeks post SCI (Fig. 5d). These results suggest that CRMP4 deletion has a neuroprotective effect on cells at the lesion site after SCI, thereby creating a permissive environment for the regrowth of axons in the injured spinal cord.

### Deletion of CRMP4 enhances axonal growth in injured spinal cord

**Figure 6:**
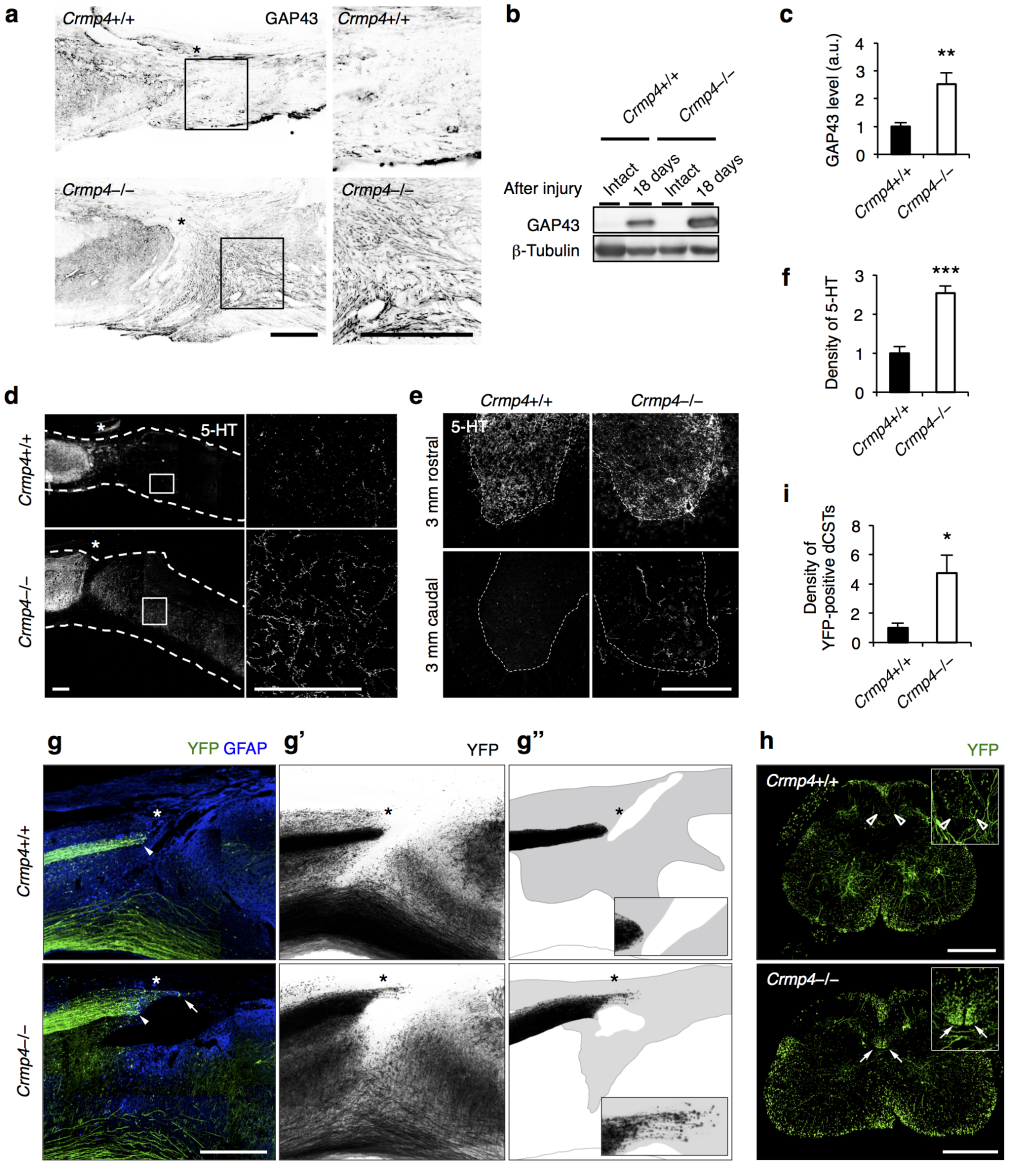
Enhanced axonal growth in *Crmp4*−/− mice after near-complete transection. (a) Representative sagittal sections of immunohistochemical analysis of GAP43-positive axons at 18 days after SCI. (b,c) Western blotting analysis with anti-GAP43 antibody (n = 6 mice for each genotype). d, days. (d) Images from sagittal sections of 5-HT immunohistochemical analysis revealed 5-HT-positive rectilinear profiles of raphespinal tract axons caudal to lesion site in *Crmp4*−/− mice at 4 weeks after SCI, while 5-HT signals were not apparent in controls. (e) Representative cross sections of spinal cords stained with anti-5-HT antibody at levels 4 mm rostral or caudal to the injury site at 4 weeks after SCI. (f) Quantitative analysis of immunoreactivity of 5-HT within 1–2 mm caudal to lesion site in parasagittal sections (n = 5 mice for each genotype). (g) Near-complete dorsal transection of spinal cord transects the main descending CST projection (solid arrowheads) at 4 weeks after SCI. Reconstructed (g′) and camera lucida drawings (g″) of YFP-labeled CST axons in all consecutive parasagittal sections. Gray areas in g″ indicate the scar tissues developing at the lesion site. The core and caudal side to lesion epicenter (asterisks) are devoid of YFP-labeled CST in *Crmp4*+/+ mice. In contrast, YFP-labeled fibers are significantly apparent within injury site along cavitation in *Crmp4*−/− mice (g; solid arrows). (h,i) Representative images of cross sections of 3 mm caudal to lesion site at 4 weeks after transection (h) and quantification of immunoreactivity of YFP-positive CST fibers at the location (i). While the absence of YFP-positive CST (open arrowheads in h) were observed in control mice, injured *Crmp4*−/− spinal cords showed a greater number of CST axons at CST location (solid arrows in h, and i, n = 4 mice for each genotype). Asterisks in images of sagittal sections indicate the lesion epicenters. The left side is rostral in all the images of parasagittal sections. *, *P* < 0.05, **, *P* < 0.01, ***, *P* < 0.001 compared with *Crmp4*+/+ controls. Statistical analysis was performed using an unpaired Student's *t* test. Data are mean ± S.E.M. Scale bars: 300 μm in a,d,e, 500 μm in g, h.

To further characterize the role of CRMP4 in limiting axonal growth *in vivo,* we performed immunohistochemical analyses of spinal cords after injury, avoiding the sections of the control spinal cords that had large cavitations, to obtain a clearer histological comparison with sections of *Crmp4*−/− spinal cords. We detected an increased density of growth-associated protein 43 (GAP43)-positive regenerating or sprouting axons caudal to the lesion site in *Crmp4*−/− mice at 18 days post SCI, while almost no expression of GAP43 in axons within and caudal to the injury site was observed in control mice (Fig. 6a). To examine which type of spinal fibers are GAP43-positive, we conducted immunostaining of GAP43 in cross sections caudal to injury site (Supplementary Fig. S4). In control mice, GAP43-positive fibers were observed only in the dorsal horn (arrowheads), which are ascending tracts from dorsal root ganglion. In contrast, *Crmp4*−/− spinal cords exhibited clear signals of GAP43 at the location of descending corticospinal tract (CST) fibers (arrows) as well as ascending tracts in the dorsal horn (arrowheads). To confirm the elevated axonal growth, we also performed immunoblotting analysis with anti-GAP43 antibody using tissue samples from intact and injured spinal cords of both groups. We detected GAP43 protein expression in the spinal cords of both groups at 18 days post SCI, but not in the intact spinal cord. We observed a significantly higher level of GAP43 protein in *Crmp4*−/− spinal tissue than in controls (Fig. 6b,c).

In this study, we utilized a 1.5-mm-depth near-complete dorsal transection model^41^ to sever the whole gray matter of the spinal cord and a group of defined pathways, including raphespinal and CST axons and all their branches, leading to hindlimb paralysis. The serotonergic (that is, 5-hydroxytryptamine [5-HT]-positive) raphespinal system contributes to locomotor circuitry and can be assessed in an anterograde fashion by simple immunohistology, since it is the only source of serotonergic input to the adult spinal cord. In control mice with dorsally transected spinal cords, 5-HT-immunoreactive fibers were observed as a few subsets caudal to the lesion site, at 4 weeks post SCI (Fig. 6d). However, in *Crmp4*−/− transected spinal tissue, a high density of 5-HT-positive fibers was detectable in parasagittal sections on the far side of the lesion (caudal to the epicenter). *Crmp4*−/− spinal cord exhibited 147.8% increase in immunoreactivity of 5-HT within 1–2 mm caudal to injury site when compared to controls (Fig. 6f, *P* < 0.05). Cross sections of spinal cord at a level 4 mm caudal to a complete transaction were also examined for 5-HT-positive profiles (Fig. 6e). As previously reported^42^, the highest density of innervations was in the ventral horn, and was observed in both groups rostral to the lesion site. 5-HT fiber density at 3 mm rostral to lesion was not different between genotypes (Fig. 6e). Although the length of serotonergic fiber at the 4-mm-caudal level was essentially undetectable in injured control spinal cord, a significant proportion of raphespinal fibers were observed in the ventral horn of the distal cord following injury in *Crmp4*−/− mice (Fig. 6e).

To examine whether deletion of CRMP4 induces CST growth, we used a yellow fluorescent protein (YFP)-expressing mouse line (YFP-H)^43^. In this transgenic mouse, corticospinal neurons in layer V of the cerebral cortex and their projecting axons in the spinal cord are strongly YFP-positive, with relatively weak YFP signal in some ascending dorsal column axons from dorsal root ganglion neurons, projections in the lateral and ventral columns, and motoneurons^43^. Because of its strong YFP signal in CST, this mouse line was used for evaluation of recovery from SCI^44^. To summarize the pattern of CST fiber growth rostral and caudal to the injury, we reconstructed all the serial microscopic images from parasagittal sections that included YFP-positive CST fibers. Proximal to the lesion at 4 weeks post SCI, the main CST appears as a tight bundle of fibers, with the labeled fibers neither entering nor growing beyond the lesion site in sagittal sections from control mice (Fig. 6g; solid arrowheads, 6g′,g″). In addition, the control mice exhibited almost no fibers in normal CST locations 3 mm caudal to the lesion site (Fig. 6h; open arrowheads). In contrast, significant numbers of YFP-positive fibers growing into the lesion scar and along cysts were observed in injured *Crmp4*−/− spinal cord (Fig. 6g; solid arrow, 6g′,g″). Moreover, we observed a higher density of YFP-positive main CST axons presenting in their normal position 3 mm caudal to the lesion in *Crmp4*−/− mice when compared to control mice (Fig. 6h; solid arrow, Fig. 6i, *P* < 0.05). Taken together, the evidence shows that deletion of CRMP4 enhanced axonal regrowth or sprouting after near-complete transection of the spinal cord.

### Locomotor recovery in *Crmp4*−/− mice after SCI

**Figure 7:**
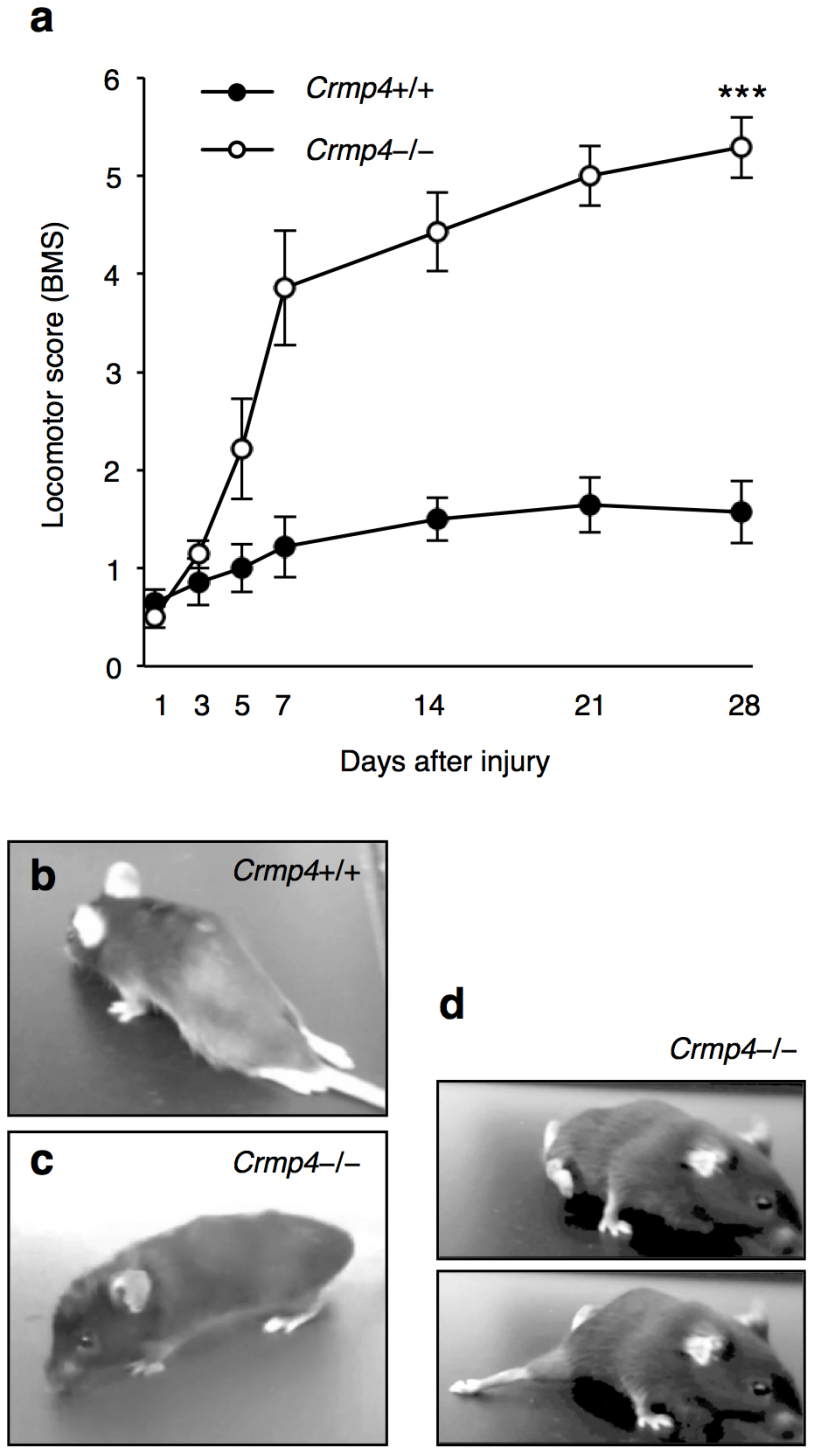
Improvement of locomotion after SCI in mice lacking CRMP4. (a) Functional analysis of open field locomotor activity by BMS scoring up to 28 days after near-complete transection of spinal cord in *Crmp4*+/+ and *Crmp4*−/− mice. Note that locomotor recovery in *Crmp4*−/− mice is significant from early stages post-SCI (n = 7 mice for each genotype). ***, *P* < 0.001, compared with scores in *Crmp4*+/+ mice. Statistical analysis was performed using the two-way ANOVA test. Data are mean ± S.E.M. (b) Photograph of a *Crmp4*+/+ mouse at 4 weeks after SCI demonstrating inability to move with hindlimbs. (c, d) Examples of *Crmp4*−/− mouse bearing its body weight at 4 weeks after SCI (c) and sweeping with the hindlimb at 1 week after SCI (d).

To examine behavioral function in *Crmp4*−/− mice after SCI, we used the Basso Mouse Scale for locomotion (BMS)^45^ to assess hindlimb motor function. In all the mice used in this study, hindlimb movement was abolished immediately after near-complete transection. At 4 weeks post SCI, hindlimb paralysis showed slight recovery in control mice (average BMS score: 1.57 ± 0.32, mean ± S.E.M., Fig. 7a,b), in agreement with results from a previous study using a near-complete-transection model in wild-type mice^41^. In contrast, the *Crmp4*−/− mice had a significantly higher BMS score (5.29 ± 0.31, mean ± S.E.M., Fig. 4a,c; Supplementary Movie 1). Notably, due to neuroprotection and reduced scar formation with CRMP4 deletion, many of these mice could move all joints of the hindlimbs freely and could support their own weight around 1 week post SCI, which is considered early for the recovery phase of an injury (Fig. 4a,d).

## Discussion

The hypothesis that CRMP4 contributes to the limitation of recovery after adult CNS trauma is supported by several major findings from this study on *Crmp4*−/− mice. First, the expression levels of pCRMP4, tCRMP4, and CRMP4b, which are suggested to contribute considerably to limiting axonal growth and to promoting cell death, are significantly increased at the lesion site in spinal cord (Fig. 1). Second, increased CRMP4 expression in activated microglia/macrophages and reactive astrocytes might contribute to secondary injury, including inflammation and scarring after spinal cord lesion (Fig. 2, 4, Supplementary Fig. S1). Third, the deletion of CRMP4 has neuroprotective effects including preservation of microtubule polymerization, cell survival, delayed demyelination, and tissue sparing. This leads to axonal growth and behavioral recovery after SCI (Fig. 3, 5, 6, 7, Supplementary Fig. S2, S3). Although cytoskeletal dynamics is commonly involved in several axon-inhibitory responses as well as in the key glial processes during inflammation and scarring^11^,^12^,^13^,^14^, no target molecule has been characterized for the missing link between axon formation and scarring. The current study demonstrates that CRMP4 is a uniquely potent factor for preventing axonal regrowth after SCI through its inhibitory and toxic effects on neurons as well as through its inflammatory effects on reactive astrocytes and microglia/macrophages.

There are several possible explanations for the evident functional recovery at an early stage after SCI in *Crmp4*−/− mice (Fig. 7). First, CRMP4 deletion could diminish the convergent signals from post SCI extracellular inhibitory factors in controlling cytoskeletal dynamics in axons. The involvement of CRMP4 in axonal inhibitory responses *in vitro* has been previously described^24^,^25^,^26^. For instance, we have shown that Myelin-associated glycoprotein-induced growth cone collapse and axonal outgrowth inhibition are significantly reduced in cultured dorsal root ganglion neurons from *Crmp4*−/− mice^24^. Moreover, a previous *in vitro* study demonstrated that CRMP4b physically and functionally interacts with RhoA in a MAI-dependent manner, leading to inhibition of neurite outgrowth^25^. However, the role of CRMP4 in CNS injuries *in vivo* was until now largely unknown. Here, we detect immunoreactivity for CRMP4 co-localized with a neuronal marker in intact and injured spinal cord (Fig. 2a,b), supported by previous studies showing CRMP4 expression in motoneurons in the mutant SOD1 mouse model^31^ and after sciatic nerve injury^32^. Moreover, we observe that CRMP4b protein expression is increased in injured axons at 2 h post SCI and that deletion of CRMP4 restored microtubule polymerization at this time point (Fig. 1a,g, Fig. 3). A previous report showed that RhoA was activated in neurons and glial cells in the white matter surrounding the injury site at the same time point post SCI^46^. Our findings thus support an inhibitory function for the RhoA-CRMP4b complex in the injured spinal cord. Moreover, Sema3A-induced phosphorylation of CRMP4 induces failure of axonal formation and elongation via disruption of CRMP4 binding to microtubules and actin^19^,^21^. It has been reported that Wallerian degeneration is mediated by CRMP phosphorylation^47^. We detected elevation of CRMP4 phosphorylation levels after SCI (Fig. 1a,b,f); it is thus possible that phosphorylated forms of CRMP4 also contribute to cytoskeletal degradation during axonal degeneration after SCI. Indeed, *Crmp4*−/− exhibited a suppression of microtubule destabilization and white matter degeneration after spinal cord transection (Fig. 3, 5d, Supplementary Fig. S2). Additionally, our preliminary experiment showed the reduction of inhibitory response to CSPG in *Crmp4*−/− cultured dorsal root ganglion neurons when compared to *Crmp4*+/+ control (unpublished observation by R.T, J. N. and T. O.). These results imply that in this study, CRMP4 deletion contributes to axonal extension by blocking its mediation of MAIs, Sema3A and CSPG-induced growth cone collapse pathways.

Next, CRMP4 deletion had neuroprotective effects on neurons after traumatic lesion, such as decreased apoptosis and neuron loss (Fig. 5a–c), leading to sparing of tissue (Supplementary Fig. S2, S3). Calpain is activated in injured spinal cord from a few minutes to several hours post SCI, and is found to induce cell death in motor neurons^48^,^49^. Activated calpain truncates CRMP4 at the C-terminus and produces a 58-kDa form called tCRMP4. The latter induces neuronal apoptosis after both *in vitro* neurotoxin treatment and *in vivo* acute traumatic brain injury^28^,^29^. We detected immediate upregulation of tCRMP4 post SCI (Fig. 1a,e) and observed a significant decrease in the number of apoptotic cells and neurons lost after spinal cord transection in the mutant (Fig. 5a–c), suggesting that deletion of tCRMP4 suppresses cell death after SCI.

Finally, we observed an early functional recovery in the mutant possibly due to reduced acute inflammatory responses and inhibition of scarring (Fig. 4 and Supplementary Fig. 1). Activation of microglia/macrophages, which causes production of proinflammatory cytokines and neurotoxic molecules, is implicated in secondary injury^35^. Reactive astrocytes are the major cellular component of the glial scar, considered a physical and chemical barrier to CNS regeneration and producing several classes of growth inhibiting molecules^2^. It has been reported that the overexpression of intrinsic anti-inflammatory molecule induced locomotor recovery within 1–2 weeks after SCI, which is an early phase of injury^50^. The initiation of inflammatory processes might be triggered by the release of toxic molecules from degenerating axons^51^,^52^. Therefore, the reduction of the inflammatory response at 1 week post SCI in *Crmp4*−/− (Fig. 4a–d) may have been caused by suppression of microtubule disassembly leading to reduction of axonal degeneration in a non-cell autonomous manner. Nevertheless, a recent *in vitro* study demonstrated that CRMP4 mediates the migratory and phagocytic capability of activated microglia via CRMP binding to F-actin^53^. We observed a dramatic reduction of non-traumatic inflammation in *Crmp4*−/− spinal cord (Supplementary Fig. S1) and an upregulation of CRMP4 in activated microglia/macrophages and reactive astrocytes after SCI (Fig. 2c,d), suggesting the possibility that CRMP4 deletion suppresses inflammation in a cell-autonomous manner *in vivo*. Although further investigation is needed to clarify the molecular mechanisms of the role of CRMP4 in inflammation, our results strongly suggest that a marked reduction of inflammation in this model due to deletion of CRMP4 (Fig. 4a–d, Supplementary Fig. S1), leading to drastic inhibition of scar formation (Fig. 4e–g), contributes to early locomotor recovery post SCI (Fig. 7).

We observed that deletion of CRMP4 enhanced immunoreactivities to GAP43 and 5-HT far caudal to the lesion epicenter, suggesting long-distance regrowth, or sprouting of serotonergic raphespinal fibers (Fig. 6a–e). We also identified CST axons using YFP-H mice^43^ where CSTs are clearly visualized as previously described^44^. While YFP-positive main dorsal CSTs disappeared within and caudal to the lesion site at 4 weeks post SCI in control mice, the genetic deletion of *Crmp4* promoted extensive regrowth or sprouting of a subset of CST axons within and into the distal (caudal) side of the lesion site (Fig. 6f,g). Distally, we observed more apparent YFP signals at the normal position of the main dorsal CST in *Crmp4*−/− mice than in the controls (Fig. 6g), suggesting that these results were due to sprouting of neighboring spared axon terminals rather than regeneration of transected axons. This conclusion seems to be strongly supported by the observed massive decrease of microtubule destabilization (Fig. 3), the decrease in secondary tissue injuries (Fig. 4,5a,5b), and the sparing of tissue (Fig. 5c,d, Supplementary Fig. S2, S3). However, it is particularly challenging to distinguish regeneration from sprouting and to detect inadvertently spared axons, because the CST axons descend in several different tracts^3^. Combinatorial modulations of both intrinsic neuronal mechanisms and extrinsic mechanisms after CNS injuries are required for long-distance axonal regeneration or sprouting and marked functional recovery after SCI^10^,^54^. Our results showed that the significant early locomotor recovery and long-distance axonal regrowth or sprouting caused by CRMP4 deletion were achieved by reducing cytoskeletal destabilization in axons and by reducing inflammatory responses, the latter leading to limited scar formation.

From a clinical perspective, it is desirable to target molecules with minimal side effects. For instance, the high lethality of *Sema3A*−/− mice raises concerns over possible strong side effects of targeting Sema3A^55^. CRMP4 has been shown to be highly expressed in the nervous system^20^. Our present results show expression of CRMP4 in neurons, activated microglia/macrophages, and reactive astrocytes, but not in these cells in a resting state. Moreover, we observe that mice lacking CRMP4 show no gross changes in body growth or fertility. These data suggest that altering CRMP4 expression may have minimal side effects on other organs when compared to targeting ubiquitous molecules such as RhoA. Thus, CRMP4 could be a candidate target in the development of neuroregenerative medicines. A peptide construct that expresses the unique N-terminal domain of CRMP4b (C4RIP– CRMP4b–RhoA inhibitory peptide) would be advantageous for use in the complex inhibitory environment prevailing after CNS injury *in vivo*^25^,^26^. While the therapeutic potential of stem/progenitor cells in cell replacement strategies for SCI has been reported^56^, numerous obstacles presented by the surrounding environment, such as secondary tissue damage and the expression of inhibitory molecules associated with scarring, still need to be overcome. Therefore, future directions should focus on the concurrent regulation of CRMP4 and other treatments/therapeutic modalities such as cell transplantation or neurotrophic factors^57^.

In conclusion, the present study demonstrates that deletion of a single protein—CRMP4—resulted in the reduction of axonal outgrowth inhibition in neurons, inflammatory responses of glia, and scarring responses of glia, thereby promoting axonal growth and functional recovery after SCI. CRMP4 may be a possible therapeutic target for the treatment of human patients with SCI.

## Methods

### Animals

The mice used in the experiments were housed in accordance with the technical protocols for animal experiments approved by the Institutional Animal Care and Use Committee at Waseda University (2013-A085). *Crmp4*−/− mice were generated and maintained in 129/Sv × C57BL/6J hybrid background as previously described^30^. The control *Crmp4+/+* mice and *Crmp4*−/− mice were obtained by intercrossing *Crmp4*+/− mice and their offspring. YFP-H mice (Jackson laboratory, ME, USA)^43^ were crossed with *Crmp4*−/− mice for the analysis of corticospinal tract axons. All the experimenters were blinded to the genotype and treatment condition.

### Surgical procedures

All surgical procedures and postoperative care were performed in accordance with guidelines of Waseda University. Mice (6–9 weeks old) were utilized for all experiments. Mice were deeply anesthetized with 2–4% isoflurane (DS Pharma Animal Health) using an inhalation anesthesia apparatus (KN-1071 NARCOBIT-E (II), Natsume Seisakusho). Laminectomies were performed at 7–8th thoracic spinal cord levels (T7–T8), exposing the spinal cord. A 1.5-mm-deep near-complete dorsal transection^41^ was performed at T7–T8 using a pair of microscissors (NAPOX® MB-50-15, Natsume Seisakusho) and the tip of a 28-gauge needle to sever dorsal white matter, all the gray matter, the 5-HT-positive raphespinal tract, and the main dorsal CST. The skin on the back was closed with a nylon stitch (USP4-0, JIS No. 2, Natsume Seisakusho). After the operation, the mice were kept warm, placed on beds of sawdust, and given manual bladder evacuation once per day. Food was provided on the cage floor, and the mice had no difficulty, accessing water. The mice that showed 0–1 points of BMS score at 1 day after SCI were used for the following experiments and analyses.

### Immunoblotting

The tissue sampling and Western blotting were performed as previously described^9^,^58^ with some modifications. A 3-mm length of spinal cord tissue centered on the injury site or injection site were dissected out at 2 and 24 h after SCI from male and female WT C57BL/6J mice, and at 1, 2, 3, 4 and 14 weeks after SCI from female WT C57BL/6J mice, and intact female WT C57BL/6J mice (n = 3 mice for each time point). The tissue samples were homogenized in lysis buffer [20 mM Tris-HCl, 150 mM NaCl, 1 mM EDTA, 10 mM NaF, 1 mM Na_3_VO_4_, 1% Nonidet P-40, proteinase inhibitor (Complete proteinase inhibitor cocktail, 11 873 580 001, Roche), and phosphatase inhibitor (PhosphoStop, 04 906 845 001, Roche)] as previously described (Ohshima et al., 2007). The homogenates were centrifuged at 1,200 rpm for 15 minutes (min) at 4 degrees. The supernatants were assayed for protein concentration using Bio-Rad Protein Assay Dye Reagent Concentrate (500-0006, Bio-Rad Laboratories) and a spectrophotometer (Gene Quant 1300, GE Healthcare). The proteins were separated on 12.5% or 15% sodium dodecyl sulfate (SDS)-PAGE gels and then transferred to Immobilon®-P Transfer Membranes (IPVH07850, Millipore). After the transfer, the membranes were placed in tris-buffered saline (TBS) with 0.05% Polyoxyethylene (20) Sorbitan Monolaurate (Tween 20) (166-21115, Wako) (0.05% TBST) containing 3% bovine serum albumin (BSA) (A7906, Sigma-Aldrich) overnight (O/N) at 4 degrees to block nonspecific binding. The blots were incubated for 1 h with following primary antibodies: anti-CRMP4 (rabbit IgG, 1:1000, AB5454, Millipore), anti-CRMP4b (rabbit IgG, kindly provided by Dr. Quinn), anti-phosphorylated (at Ser 522) CRMP4 (rabbit IgG, 1:1000)^30^, anti-α-tubulin (rabbit IgG, 1:1000, T6199, Sigma-Aldrich), anti-GAP43 (rabbit IgG, 1:1000, ab16053, Abcam), anti-β-tubulin (rabbit IgG, 1:1000, T8328, Sigma-Aldrich), anti-TNFα (rabbit IgG, AB2148P, Millipore), diluted in blocking buffer at room temperature (RT). The membranes were then washed in 0.05% TBST three times for 10 min and incubated with horseradish peroxidase-conjugated anti-mouse or anti-rabbit IgG (1:10,000, sc-2005 or sc-2004, Santa Cruz Biotechnology) at RT for 1 h. After washing in 0.05% TBST three times for 10 min, the membranes were developed using a color substrate. (Pierce® Western Blotting Substrate Plus, NCI32132JP, Thermo Fisher Scientific). The reaction was stopped by washing each membrane with TBS. Tubulin was detected in parallel as loading controls. The membranes were scanned with a Luminescent Image Analyzer (LAS-3000, Fujifilm), and the resulting digital images were quantified and normalized relative to tubulin level for each sample using the ImageJ software (National Institutes of Health).

### Immunohistochemistry

The mice were perfused after injury with ice-cold 4% paraformaldehyde/PBS solution. A 15-mm length of spinal cord centered on the injury site or injection site was dissected out and postfixed O/N in the same fixative. Then the spinal cord was soaked in a succession of solutions at 4 degrees O/N (PBS, 10% sucrose and 20% sucrose, respectively) and embedded in a 2:1 mixture of Tissue Tek® optimal cutting temperature (OCT) compound (4583, Sakura Finetek) and 20% sucrose for cryostat sectioning (30 μm) on a Cryostat (CM1860, Leica). The sections were mounted on MAS-coated glass slides (S9441, Matsunami) and stored at −20 degrees until further analysis. We performed immunofluorescence staining was performed as previously described (Ohshima et al., 2007). Prior to staining, tissue sections were dried in air for 1 h and washed in PBS for 30 min followed by permeabilizing and blocking with 3% normal horse serum (HS, S-2000, Vector Laboratories) diluted with 0.1% Polyoxyethylene (10) Octylphenyl Esther (Triton X-100, 169-21105, Wako)/PBS (PBST) for 1 h. Incubation at 4 degrees O/N was carried out with the following primary antibodies; anti-CRMP4 (rabbit IgG, 1:200, AB5454, Millipore), anti-MAP2 (mouse IgG, 1:200, MAB3418, Millipore), anti-detyrosinated α-tubulin (Glu-tubulin) (rabbit IgG, 1:500, ab24622, Abcam), anti-neuron-specific class III β-tubulin (Tuj1, mouse IgG, 1:1000, MMS-435P, Covance), anti-GFAP (mouse IgG, 1:400, G3893, Sigma-Aldrich), anti-OX-41 (mouse IgG, 1:200, MAB1407P, Millipore), anti-Iba1 (rabbit IgG, 1:500, 019-19741, Wako) and anti-GFAP (rabbit IgG, 1:500, Z0334, Dako) antibodies. After being washed in 0.01% PBST (three times, 10 min), sections were incubated with the secondary antibodies [Alexa Fluor® 488 or 568 goat anti-rabbit IgG (H + L) or Alexa Fluor® 594 goat anti-mouse IgG (H + L), 1:1000, A11008, A11011, A11005, Invitrogen, and NeuroTrace® 530/615 red fluorescent Nissl stain, 1:250, N-21482, Molecular Probes] in 0.01% PBST for 1 h at RT. Finally, the slides were washed three times for 10 min in 0.01% PBST and once in PBS for 1 min before being dried and coverslipped. For immunostaining with anti-GAP43 (rabbit IgG, 1:200, ab16053, Abcam), and anti-collagen IV antibody (1:200, ab19808, Abcam), the sections were blocked with 5% HS in PBS for 1 h and permeabilized with 0.2% Tween 20 in PBS four times for 5 min and 5% HS in 0.1% PBST for 1 h. For immunostaining with anti-5-HT (rabbit IgG, 1:4000, 20080, Immunostar), tissue sections were washed in high-salt buffer (HSB; 500 mM NaCl, 9.2 mM NaH_2_PO_4_, 12.5 mM Na_2_HPO_4_) three times for 10 min and blocked with 5% HS diluted with 0.3% Triton X-100 in HSB. After primary and secondary antibody reaction, the slides were washed three times for 10 min with HSB. Terminal deoxynucleotidyl transferase (TdT)-mediated deoxyuridine triphosphate-biotin nick end labeling (TUNEL) using Apop Tag Red (S7165, Millipore) was performed to detect apoptotic cells in the transected spinal cord, following manufacturer's protocol. FluoroMyelin™ Red Fluorescent Myelin Stain (F34652, Molecular Probes) was used to reveal myelin distributions in the transected spinal cord, following manufacturer's protocol. Nuclear staining was performed with anti-fade mounting media Vectorshield® Mounting Medium for Fluorescence with DAPI (H-1200, Vector Laboratories) in identical sections. Images were visualized using microscopy (BX51, Olympus and BZ 8100, Keyence) and confocal fluorescence microscopy (FV-1000, Olympus).

### Quantification for immunohistochemistry

To analyze Glu-tubulin distribution in the axons, we measured the length of Glu-tubulin-positive and Tuj1-positive fibers in each section of the spinal cords as previously described in detail^34^. Briefly, the area of dorsal white matter in the rostral and caudal stump, ~3 mm rostrally from the injury site, was studied. The average length of a subset of fibers was calculated, and the data was normalized to the percentage of control. For each experimental condition, four random images from each of the three independent sections from three mice were captured and 3 sections containing at least 20 axons per mouse were analyzed. The lesion volume was delineated by its bounding surface, which is defined by a series of closed contours in the serial sections. For the quantification, 2–4 serial cross sections taken every 300 μm or serial parasagittal sections taken every 180 μm sections were analyzed as previously described^59^ with some modifications. The areas of the microglial and astroglial activation, collagen IV-positive scar and the immunoreactivity of GFAP and 5-HTwas quantified with NIH ImageJ software (National Institutes of Health). For analysis of inflammatory responses after SCI, images of cross sections at 1.5 mm caudal to lesion site were analyzed. Zymosan A-induced inflammatory responses were examined 750 μm rostral and caudal to the injection site. Counts of the numbers of TUNEL-positive apoptotic cells with DAPI-positive nuclei were performed within areas both 2.5 mm rostral and 2.5 mm caudal to the epicenter of injury. Quantification of 5-HT immunoreactivity was analyzed within 1–2 mm caudal to lesion epicenter of parasagittal sections.

### Microinjections

Non-traumatic intraspinal microinjection of Zymosan A was performed as previously described^60^. Briefly, 50 nL Zymosan A (12.5 mg/ml, 273-01491, Wako) or 0.1 M PBS were injected into the lateral funiculi of spinal cords 1–1.2 mm lateral to the spinal cord midline and 0.5–0.7 mm deep at the level of T7/8. Injections were carried out over 5 min using calibrated pressure ejection (Harvard Apparatus, Pump 11 Elite Syringe Pumps). Mice were sacrificed at 3 days after injection and then analyzed.

### Behavioral analysis

Hindlimb motor function was evaluated 1, 3, 5, 7, 14, 21 and 28 days after injury (n = 7 per group) using the locomotor rating of the Basso Mouse Scale (BMS) as previously described in detail^45^. This scale ranges from 0, indicating complete paralysis, to 9, indicating normal movement of the hindlimbs. A team of two experienced examiners evaluated each animal for 3–5 min and assigned a score based on a defined method where performance of the left and right hindlimbs was averaged.

### Statistical analysis

Statistical differences between two groups were calculated with an unpaired two-tailed Student's *t* test. Behavioral analysis was performed using two-way analysis of variance (ANOVA), and the rest of the data was analyzed using one-way ANOVA followed by Dunnett's or Tukey's *post-hoc* multiple-comparison test as appropriate to the design. The variance similarity between two samples was confirmed using F-test. All analyses were conducted using GraphPad Prism software version 6.0b.

## Author Contributions

J.N. and T.O. designed experiments and interpreted data. J.N., Y.K., K.O. and T.O. developed the SCI model and performed the biochemical, the histological and the behavioral experiments. K.T. taught J.N., Y.K., K.O. the techniques necessary for the SCI model. N.Y. and Y.G. jointly directed the project. J.N. and T.O. wrote the paper with input from all co-authors.

## Supplementary Material

Supplementary Information Supplementary information

Supplementary Information Supplementary Movie 1
