# Supplementary material for: Crmp4 deletion promotes recovery from spinal cord injury by neuroprotection and limited scar formation
Source: Sci Rep. 2015 Feb 5;5:8269. doi: 10.1038/srep08269 (PMC4317684; doi:10.1038/srep08269)
Supplement: Supplementary Information [file srep08269-s1.pdf]

Supplementary Information

"*Crmp4* deletion promotes recovery from spinal cord injury
by neuroprotection and limited scar formation"

Jun Nagai[1,2], Yoshiteru Kitamura[1], Kazuki Owada[1], Naoya Yamashita[3], Kohtaro Takei[4], Yoshio Goshima[3] and Toshio Ohshima[1*]

[1] *Department of Life Science and Medical Bioscience, Graduate School of Advanced Science and Engineering, Waseda University, Tokyo, 162-8480 Japan*

[2] *Research Fellow of Japan Society for the Promotion of Science*

[3] *Department of Molecular Pharmacology and Neurobiology, Graduate School of Medicine, Yokohama City University, Yokohama 236-0004, Japan*

[4] *Department of Medical Life Science, Graduate School of Medical Life Science, Yokohama City University, Yokohama, 236-0004, Japan.*

* Corresponding author: Toshio Ohshima
Department of Life Science and Medical Bioscience, Waseda University,
2-2 Wakamatsu-cho Shinjuku-ku, Tokyo 162-8480, Japan
Tel: -81-3-5369-7321, Fax: -81-3-5369-7302, e-mail: ohshima@waseda.jp

Supplementary Fig. S1 Nagai et al.

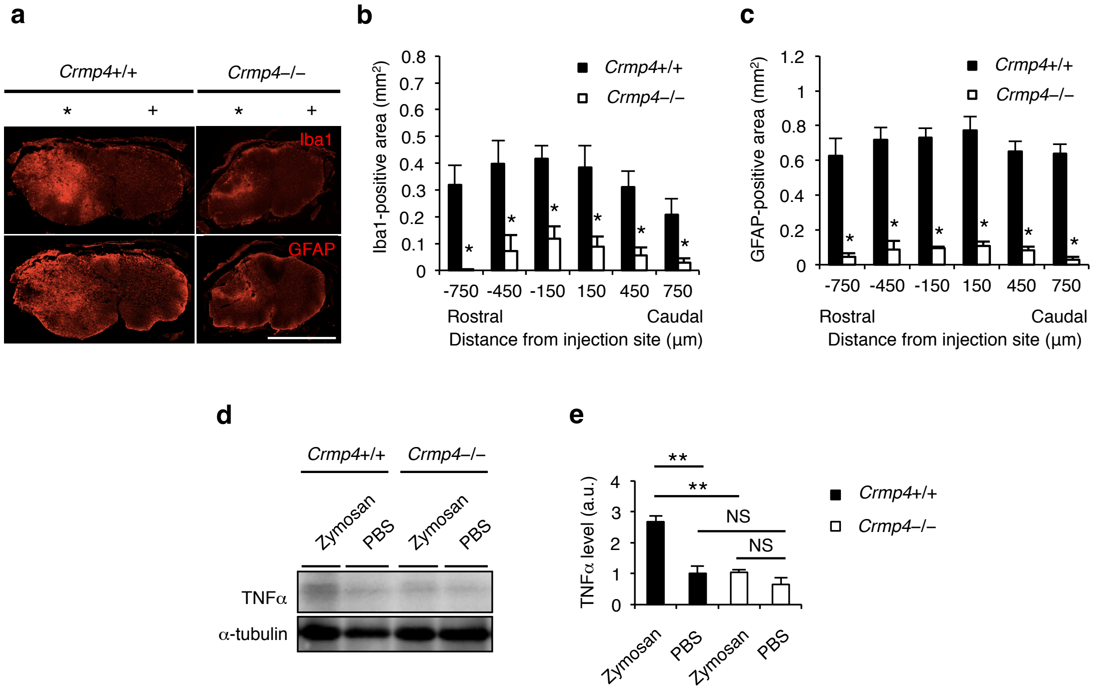

Supplementary Fig. S2 Nagai et al.

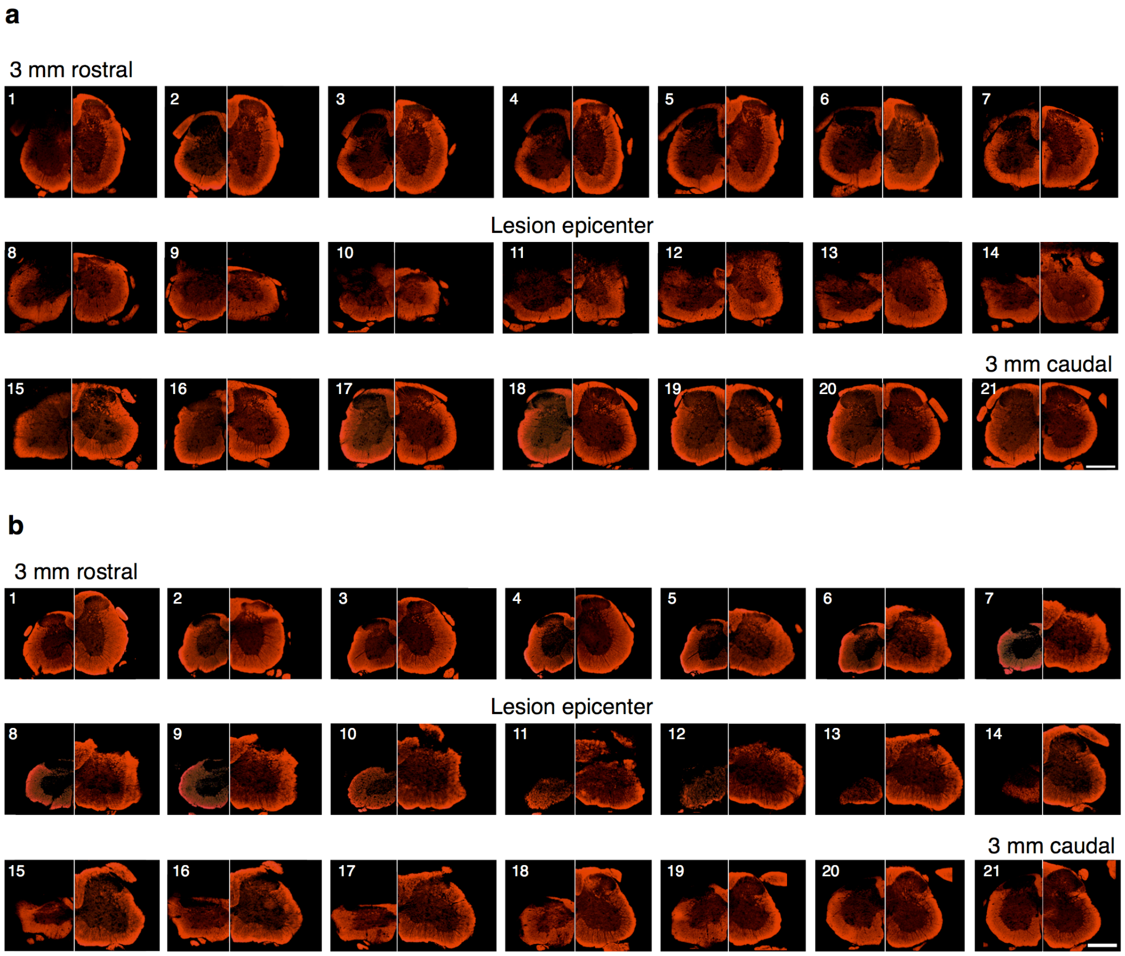

Supplementary Fig. S3 Nagai et al.

a

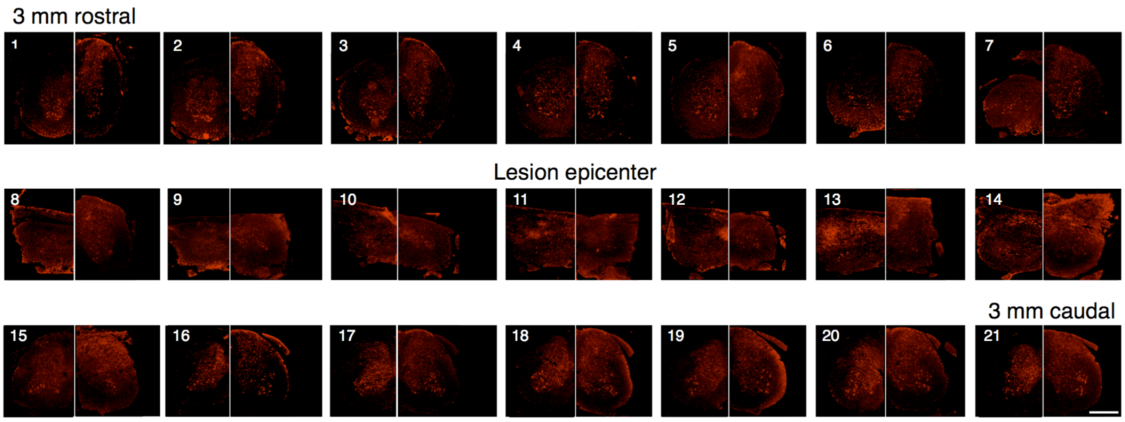

b

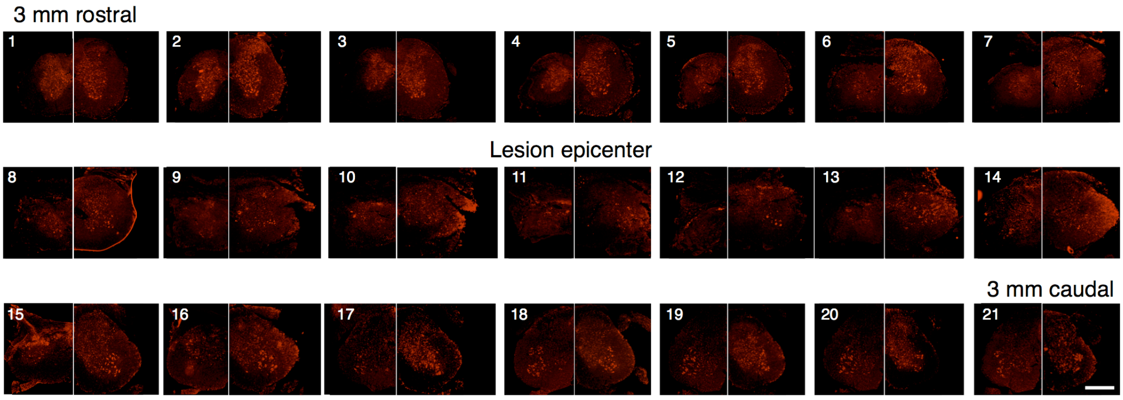

Supplementary Fig. S4 Nagai et al.

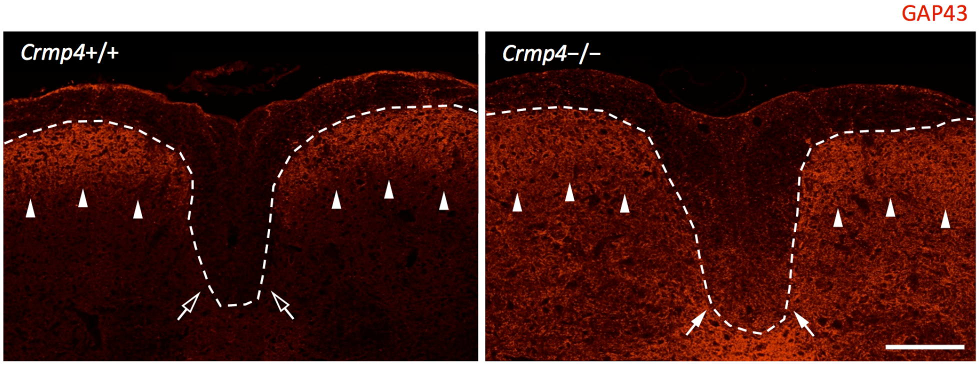

Supplementary Figure 1. CRMP4 deletion reduces non-traumatic inflammation. (**a**) Representative transverse images of thoracic spinal cords from mice with CRMP4 deletion stained with anti-Iba1 (marker for microglia/macrophages) and anti-GFAP (marker for astrocytes*)* antibodies at 3 days post induction of non-traumatic inflammation by Zymosan A. *, Zymosan A injection site; +, PBS injection site. Intraspinal micropipette insertion is confirmed to be non-traumatic by failure of PBS microinjection to activate microglia and astroglia. Scale bars = 1000 μm. (**b, c**) Areas of quantified microglial and astroglial activation in transverse sections taken every 300 μm around the injection site. n = 3. *, $P < 0.05$ compared with *Crmp4*+/+ controls. Statistical analysis by unpaired Student's t test. (**d, e**) Western blotting analysis with anti-Tumor necrosis factor alpha antibody after Zymosan A- or buffer-injection of spinal cords (n = 3 animals). **, $P < 0.01$. Statistical analysis by one-way ANOVA followed by Tukey's multiple comparison test. Data are mean ± S.E.M. PBS, phosphate-buffered saline; TNFα, Tumor necrosis factor alpha; NS, not significant.

Supplementary Figure 2. Serial cross sections from *Crmp4*+/+ and *Crmp4*−/− mice after SCI with myelin staining. Myelin staining of serial cross sections taken every 300 μm reveals demyelination through the lesion epicenter (#11). Sections #1–10 are rostral and #12–21 are caudal to the injury site. The left half of each spinal cord section illustrated is from a *Crmp4*+/+ mouse and the right half is from a *Crmp4*−/− mouse. The areas of myelin-positive white matter are not different among samples at 1 week post SCI, indicating that the dorsal transections to the animals were of uniform extent (**a**). However, at 4 weeks post injury, *Crmp4*−/− spinal cord exhibits significantly increased sparing of tissue, including white matter, when compared with control (**b**). The images of #11 and #14 are shown in Fig. 5d. Scale bar: 500 μm.

Supplementary Figure 3. Serial cross sections from *Crmp4*+/+ and *Crmp4*−/− mice after SCI with Nissl staining. Nissl staining of serial cross sections taken every 300 μm reveals demyelination through the lesion epicenter (#11). Sections #1–10 are rostral and #12–21 are caudal to the injury site. The left halves of the spinal cord sections are from a *Crmp4*+/+ mouse and the right halves are from a *Crmp4*−/− mouse at 1 week post injury (**a**) or at 4 weeks post injury (**b**). The images of #11 and #16 are shown in Fig. 5c. Scale bar: 500 μm.

Supplementary Figure S4. GAP43-immunolabeling in cross sections of injured spinal cords from *Crmp4+/+* and *Crmp4−/−* mice. Axonal growth in cross sections at 1.5 mm caudal to lesion site at 4 weeks after injury was detected by immunostaining with anti-GAP43 antibody. Although there was absent of GAP43-positive fibers at the location of descending corticospinal tracts in a control spinal cord (open arrows), GAP43 signals were apparent there in a *Crmp4*−/− spinal cord (arrows). Ascending fibers from dorsal root ganglion were GAP43-positive in both genotypes (arrowheads). Scale bar: 100 μm.

Supplementary Movie 1. Locomotor recovery after spinal cord injury in mice lacking CRMP4. Example of a *Crmp4*+/+ mouse demonstrating inability to move with hindlimbs and a *Crmp4*−/− mouse bearing its body weight at 4 weeks after spinal cord injury.
